# Analytical performance of aPROMISE: automated anatomic contextualization, detection, and quantification of [^18^F]DCFPyL (PSMA) imaging for standardized reporting

**DOI:** 10.1007/s00259-021-05497-8

**Published:** 2021-08-31

**Authors:** Kerstin Johnsson, Johan Brynolfsson, Hannicka Sahlstedt, Nicholas G. Nickols, Matthew Rettig, Stephan Probst, Michael J. Morris, Anders Bjartell, Mathias Eiber, Aseem Anand

**Affiliations:** 1 Department of Data Science and Machine Learning, EXINI Diagnostics AB, Lund, Sweden; 2 Radiation Oncology Service, VA Greater Los Angeles Healthcare System, Los Angeles, CA USA; 3 Department of Radiation Oncology, David Geffen School of Medicine, University of California Los Angeles, Los Angeles, CA USA; 4 Department of Urology, David Geffen School of Medicine, University of California Los Angeles, Los Angeles, CA USA; 5 Institute of Urologic Oncology, Jonsson Comprehensive Cancer Center, University of California Los Angeles, Los Angeles, CA USA; 6 Division of Hematology-Oncology, Greater Los Angeles Healthcare System, Los Angeles, CA USA; 7 Nuclear Medicine, Medical Imaging, Jewish General Hospital, McGill University, Montreal, QC Canada; 8 Department of Medicine, Memorial Sloan Kettering Cancer Center, New York, NY USA; 9 Weill Cornell Medical College, New York, NY USA; 10 Department of Translational Medicine, Division of Urological Cancers, Lund University, Lund, Sweden; 11 Department of Nuclear Medicine, Klinikum Rechts Der Isar, Technical University of Munich, Munich, Germany

**Keywords:** aPROMISE, Segmentation, Standardized reporting, PSMA PET/CT evaluation

## Abstract

**Purpose:**

The application of automated image analyses could improve and facilitate standardization and consistency of quantification in [^18^F]DCFPyL (PSMA) PET/CT scans. In the current study, we analytically validated aPROMISE, a software as a medical device that segments organs in low-dose CT images with deep learning, and subsequently detects and quantifies potential pathological lesions in PSMA PET/CT.

**Methods:**

To evaluate the deep learning algorithm, the automated segmentations of the low-dose CT component of PSMA PET/CT scans from 20 patients were compared to manual segmentations. Dice scores were used to quantify the similarities between the automated and manual segmentations. Next, the automated quantification of tracer uptake in the reference organs and detection and pre-segmentation of potential lesions were evaluated in 339 patients with prostate cancer, who were all enrolled in the phase II/III OSPREY study. Three nuclear medicine physicians performed the retrospective independent reads of OSPREY images with aPROMISE. Quantitative consistency was assessed by the pairwise Pearson correlations and standard deviation between the readers and aPROMISE. The sensitivity of detection and pre-segmentation of potential lesions was evaluated by determining the percent of manually selected abnormal lesions that were automatically detected by aPROMISE.

**Results:**

The Dice scores for bone segmentations ranged from 0.88 to 0.95. The Dice scores of the PSMA PET/CT reference organs, thoracic aorta and liver, were 0.89 and 0.97, respectively. Dice scores of other visceral organs, including prostate, were observed to be above 0.79. The Pearson correlation for blood pool reference was higher between any manual reader and aPROMISE, than between any pair of manual readers. The standard deviations of reference organ uptake across all patients as determined by aPROMISE (SD = 0.21 blood pool and SD = 1.16 liver) were lower compared to those of the manual readers. Finally, the sensitivity of aPROMISE detection and pre-segmentation was 91.5% for regional lymph nodes, 90.6% for all lymph nodes, and 86.7% for bone in metastatic patients.

**Conclusion:**

In this analytical study, we demonstrated the segmentation accuracy of the deep learning algorithm, the consistency in quantitative assessment across multiple readers, and the high sensitivity in detecting potential lesions. The study provides a foundational framework for clinical evaluation of aPROMISE in standardized reporting of PSMA PET/CT.

**Supplementary Information:**

The online version contains supplementary material available at 10.1007/s00259-021-05497-8.

## Introduction

Prostate cancer is the most common solid tumor in men, with 1,094,916 incidence cases and 307,481 deaths estimated globally in 2012 [1]. The accurate detection of the disease and its subsequent staging are critical for selection of appropriate treatment strategies. Especially the differentiation between those with localized or regional disease who can be treated with curative intent versus those with metastatic disease is crucial. Whether or not surgery, radiation, and/or systemic treatments are appropriate for a given patient is driven in large part by the clinical stage [2]. Targeted molecular imaging with positron emission tomography/computed tomography (PET/CT) is a highly versatile imaging technology to inform staging and management decisions for patients with a variety of cancers.

In prostate cancer, PET tracers targeting prostate-specific membrane antigen (PSMA) have demonstrated high diagnostic accuracy for the detection of both regional and distant metastatic prostate cancer [3, 4]. The higher sensitivity and specificity of PSMA PET in detecting metastatic prostate cancer will have strong implications in management of patients. To demonstrate the association of PSMA imaging with clinical outcome, there is an urgent need to standardize PSMA assessment. Recent efforts in standardizing the assessment of PSMA scans have resulted in proposals for lesion characterization and reporting—EANM, PSMA-RADS, and PROMISE criteria [5–7]. While all the proposed criteria are focused on the characterization of individual PSMA lesions based on the location and the definition of significant uptake, the PROMISE standard is also proposing a patient level classification (miTNM), which is based on the total burden and its location of the disease in the PET/CT image. A recent study comparing such standardized assessments has shown that they have high inter-reader reproducibility [8].

However, the adoption and implementation of these standards in routine clinical practice is limited by the fact that the adherence to these guidelines is a manual and a labor-intensive process. The manual work can be greatly facilitated through automated image analysis. The structural radiological processes, including the segmentation of anatomical structures (from CT), can be automated to contextualize and characterize the functional imaging. Knowing the anatomical context is needed both for normal tissue reference uptake estimation as well as accurate detection of potential lesions, since uptake in the lesion as well as in the background may differ between tissues.

Deep learning organ segmentations in CT have been used in automated analyses of PSMA PET to exclude physiological uptake in certain high-uptake organs when detecting PSMA-positive lesions and estimating tumor burden. However, achieving high sensitivity while limiting the number of false positives outside these organs remains challenging. Previous lesion detection approaches for PSMA-PET [9, 10] used a liver uptake-based threshold to select possible lesions in patients with advanced prostate cancer. Such methodologies likely capture most lesions with tracer uptake more than the liver but cannot be used to detect PSMA avid disease in general, as many lesions have a SUV_max_ below the threshold of mean liver uptake. Others have presented deep learning-based methods for detection and segmentation of possible lesions [11, 12]. In automated image analysis, blob detection algorithms are commonly used to detect salient regions in images [13], and the use of such methods has the potential to capture lesions with maximal standardized uptake value (SUV) below liver uptake. Blob detection algorithms would also have the capacity to detect lesions in, e.g., uncommon locations, or with unusual uptake patterns and could also be easily extended to handle a wider range of tracers, somethings deep learning-based methods may struggle with.

An additional issue with threshold-based lesion segmentation is that in lesions with low or subtle uptake, a rigid rule of segmenting based on 50% or 30% of SUV_max_ of the lesion, would result in inaccurate over-segmentation. High uptake adjacent to lesions, for example in intestines, also confounds threshold-based segmentation. The fast marching method, used for segmentation in a wide variety of tasks [14], can be employed for lesion segmentation in this setting to avoid these problems.

To overcome the technical challenges and to assist readers in adhering to the standardized guidelines for the implementation of PSMA imaging, we have developed aPROMISE—**a**utomated **Pro**state **M**olecular **I**maging **S**tandardized **E**valuation. aPROMISE is a CE marked software as a medical device that employs deep learning technology to automate the segmentation of organs in low-dose CT images and quantifies the mean tracer uptake in the reference organs. Subsequently, aPROMISE uses blob detection and fast marching methodology to detect and segment regions of interest as potential pathological lesions in PSMA PET/CT. The intent of aPROMISE is to reduce the laborious task and to assist the readers in standardizing the PSMA imaging assessment. Therefore, in the application, it is the physicians that still must review the image and make the selection for the lesions. However, when the physician makes the call that a lesion needs to be marked as suspicious, then the technology facilitates the standardization of assessment by automating the laborious task of localization, segmentation, and quantification. The illustrative workflow has been demonstrated in supplemental Figure 1.

The aPROMISE workflow has demonstrated low inter-reader variability and high efficiency in the quantification and staging of intermediate to high-risk prostate cancer [15]. In the current study, we intend to analytically evaluate the technical performance of aPROMISE. The objective of the study is threefold: (1) to evaluate the accuracy of the automated organ segmentation applied to low-dose CT scans, (2) to evaluate the consistency of the automated quantitative tracer uptake in reference organs of PSMA PET/CT, and (3) to evaluate the sensitivity of automated detection of potential lesions in PSMA PET/CT.

## Materials and methods

### Study data and design

The study is retrospective in nature to evaluate the performance characteristics of the aPROMISE platform. The study data, the training and tuning data, and the study design are defined in detail below. The objectives and endpoint analysis for each of the three analytical studies are summarized in Table 1. The investigations that generated the PSMA PET/CT received approval from the respective local institutional review boards (detailed in sections below).

**Table 1:** The objectives and endpoint analysis for each of the three analytical studies are summarized below

Objectives	Independent validation data	Design	Endpoints
To determine the accuracy of organ segmentation deep learning algorithm on low-dose CT	PSMA PET/CT scans from investigational studies under PyL Research Access Program. IND #121064 Evaluable total *N* = 20 patients	Auto-segmentation were compared against the manually segmented organs *N* = 20	The Dice score –automated against manual segmentation
To determine the consistency of the reference organ uptake in PSMA PET	A phase 2/3 prospective multi-center study of the diagnostic accuracy of PSMA PET/CT with [^18^F]DCFPyL in patients with prostate cancer (OSPREY) NCT02981368 Evaluable total *N* = 339 patients (cohort A *N* = 250; cohort B *N* = 89)	Automated assessment of the uptake in reference organs was compared against three independent manual assessment *N* = 89 (cohort B)	The correlation and standard deviation of automated assessment against the manual standard
To determine the detection sensitivity of the candidate lesions in PSMA PET		Automated detection and segmentation of potential lesions was reviewed by three independent manual assessment *N* = 295 (cohort A-250 + cohort B*-45)	The percent of manually selected lesions that were automatically pre-selected by aPROMISE

*For lesion detection, the cohort B was restricted to patients that did not have diffused metastatic disease

### Study data

#### CT segmentation

The deep learning segmentation of low-dose CT was validated on [^18^F]DCFPyL PET/CT images from the PyL Research Access Program. The low-dose CT component of the PET/CT from 20 randomly selected patients was used to create the manual annotations (ground truth) for the organ segmentations by an experienced nuclear medicine reader. The images were obtained under the auspices of an Investigational New Drug application (IND #121064). The ethical permission for the [^18^F]DCFPyL PET/CT images under PyL Access Program was obtained from the institutional review board at Jewish General Hospital, Montreal, Canada, and from John Hopkins from the local institutional review board.

#### Reference quantification and detection in PSMA PET

The evaluation of reference quantification and detection sensitivity in PSMA PET was performed on all patients with evaluable DICOM PET/CT images from the phase 2/3 OSPREY study (Clinicaltrials.gov Identifier NCT02981368). The study enrolled two prostate cancer patient populations able to provide histopathology verification: Cohort A enrolled 252 men with newly diagnosed high-risk prostate cancer planned for radical prostatectomy (RP) with pelvic lymph node dissection (PLND), and cohort B enrolled 93 men with presumptive radiologic evidence of recurrent or metastatic prostate cancer seen on conventional imaging and considered feasible for biopsy confirmation. Of the total PET/CT images from the OSPREY study, six were unevaluable due to DICOM non-conformity and were discarded in this analytical performance study—total evaluable 339 scans, 250 in cohort A and 89 in cohort B.

The OSPREY study was conducted across 10 sites in the USA and Canada, and it was approved by the institutional review board at each participating institution. Prior to study enrollment, written informed consent was obtained from all patients. The study was conducted in accordance with the Declaration of Helsinki and the International Conference on Harmonization Guidelines for Good Clinical Practice.

### Study design

#### Automated segmentation in low-dose CT

Deep learning segmentations of 5 bone regions defined in the OSPREY study, as well as 9 soft tissue organs, were compared to the manual segmentations in full body PSMA PET/CT scans from 20 patients. All images were acquired without contrast agents. Thirteen patients were positioned with arms above head and remaining seven with arms along the body. The manual segmentation was independently performed by an experienced nuclear medicine physician.

#### Automated reference quantification in PSMA PET

Three readers independently generated blood-pool and liver reference values in all 89 patients from cohort B in OSPREY study. In the PSMA PET/CT scans, automated mediastinal blood pool and liver reference values were compared to mediastinal and liver uptake assessed with the manual method of placing volume of interest (1 cm diameter) within the descending thoracic aorta. The standard liver uptake was assessed by placing volume of interest (3 cm in diameter) within the right lower lobe of the liver.

#### Automated detection of potential lesions in PSMA PET

The performance of automated detection of potential PSMA positive local lymph lesions and bone and lymph metastasis was validated on all 250 patients from cohort A and 45 patients from cohort B, which was restricted to low tumor burden and did not contain diffuse metastatic disease. All images were read by three independent US Board certified nuclear medicine physicians through aPROMISE. All three readers were experienced in nuclear imaging and had prior experience in PSMA assessment. Two of the readers had extensive prior experience (approximately 5 years) in imaging using multiple PSMA ligands; in comparison, one reader had limited experience (approximately 1 year) with exposure to one PSMA ligand. The sensitivity of the automated detection method was evaluated as the percent of manually selected lesions that were automatically detected and pre-segmented by aPROMISE. All patients in the study had confirmed prostate cancer, and endpoints of the study were to evaluate reproducibility of calls and sensitivity of detecting lesions outside of the prostate.

### Algorithm description

#### Automated CT segmentation

**Fig. 1:**
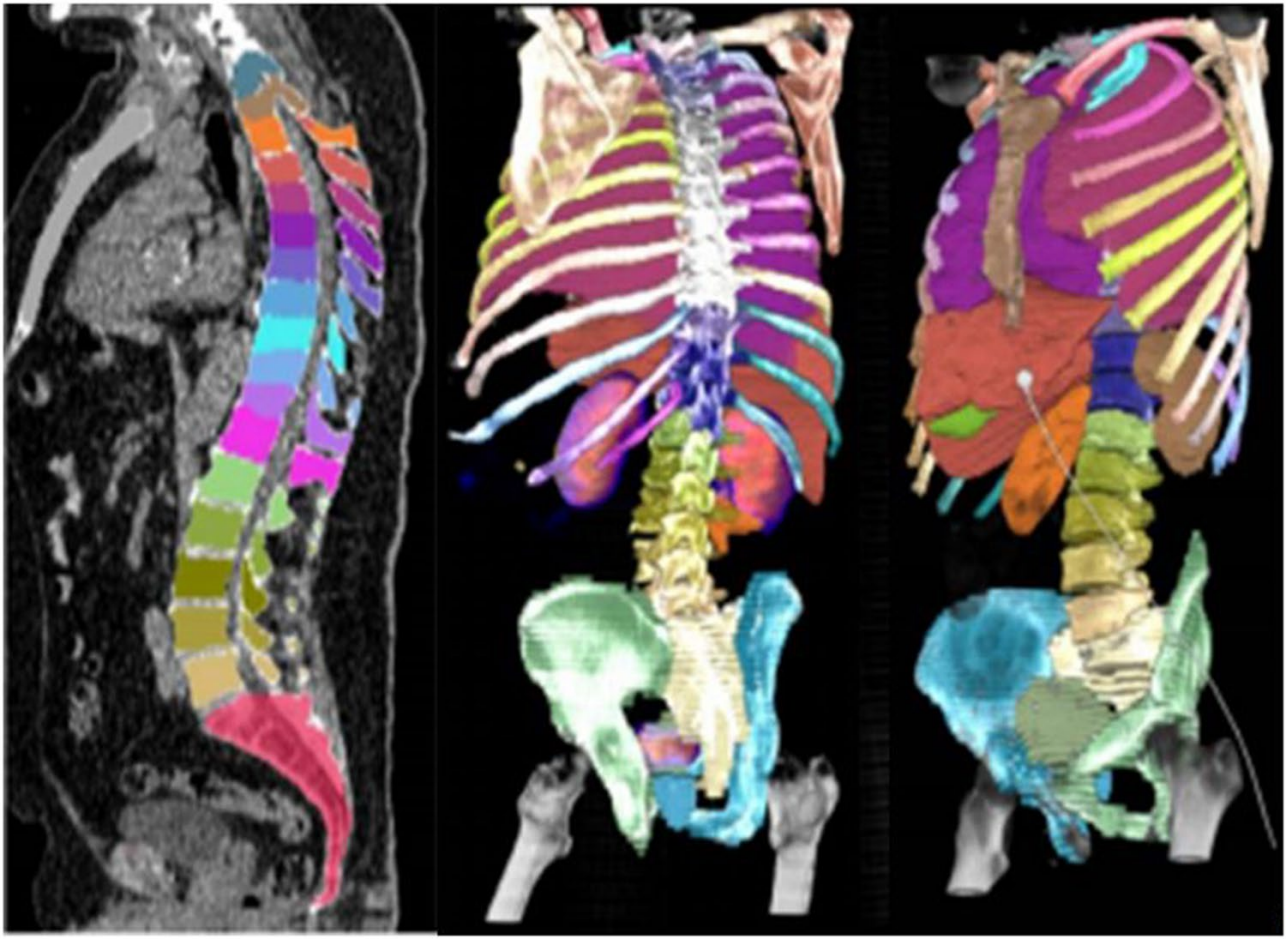
Deep learning automated segmentation of fifty-one bones and nine soft tissue organs in the low-dose CT of PSMA PET/CT

From the CT image, a cascaded deep learning pipeline based on the U-net architecture [16], segments 51 bones and 8 or 9 visceral organs, depending on whether the patient has had radical prostatectomy or not (Fig. 1). Training and tuning data for the pipeline were annotated by experienced radiologists or nuclear medicine readers and contained, in total, 246 patients (Supplemental data Table 1). For validation, the 51 bones are grouped into 5 regions defined in the OSPREY study. The training data consisted of CT images both with and without occurrence of contrast agents.

#### Automated reference organ uptake in PSMA PET

**Fig. 2:**
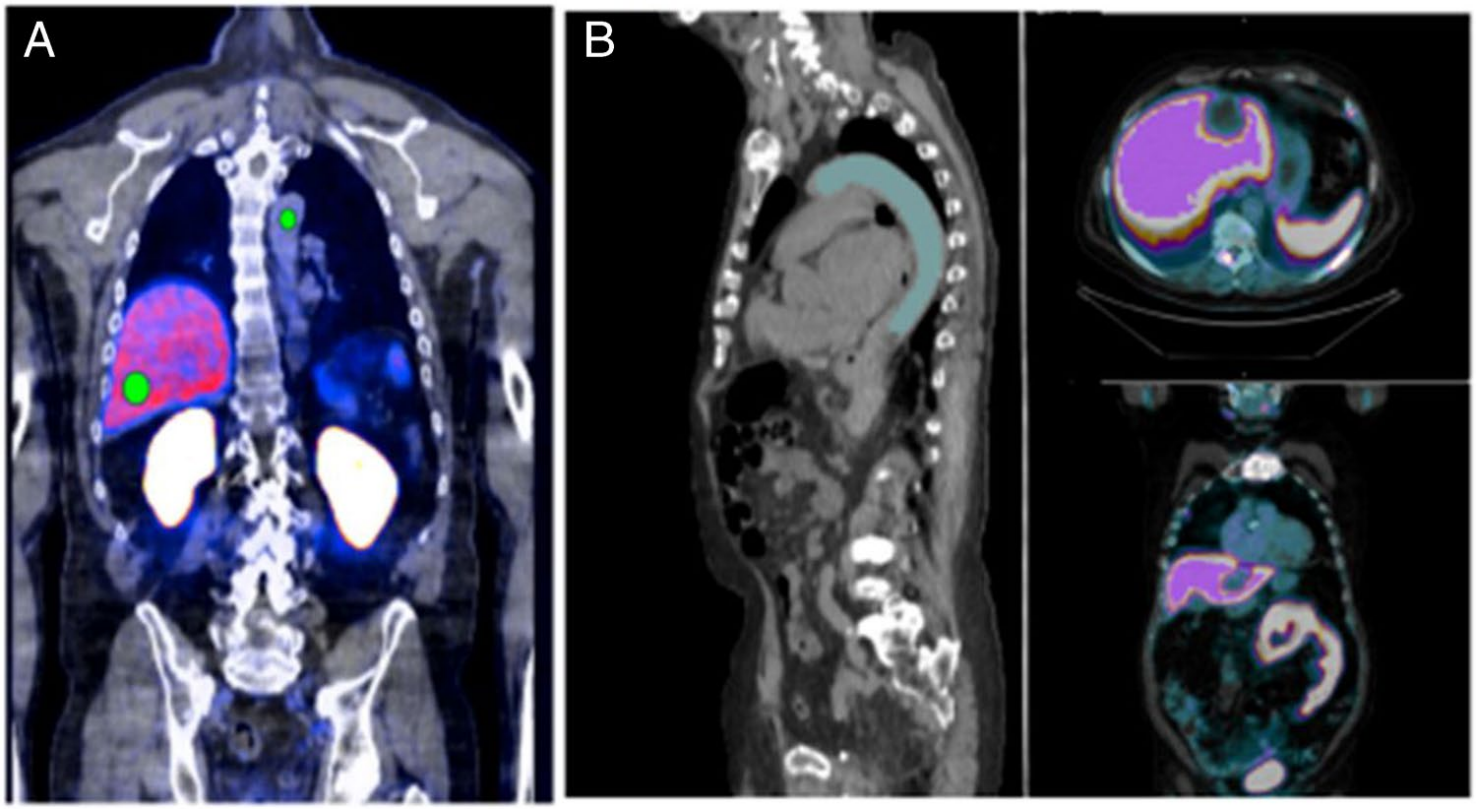
Manual placement of fixed ROI on a selected slice and location of liver and aorta to obtain SUVmean (**A**), against the automated mean reference organ uptake of liver and blood pool (aorta) (**B**) facilitated by the volumetric automated segmentation of the reference organs

The automated segmentations of the liver and aorta are eroded; i.e., the voxels close to the boundaries are removed to ensure that the segmentations are within the target organ in the PET even when there is minor misalignment. The aorta SUV reference is computed as the mean of the values in the interquartile range of the SUV within the eroded aorta segmentation. Due to breathing attenuation, the liver may contain regions with artifactually low uptake that are inappropriate to include as reference tissue. To account for this, we computed the liver reference SUV to be the largest mode in a two-component Gaussian Mixture Model fitted to the intensities in the liver segmentation. An illustrative example of automated segmentation of reference organ to compute the mean tracer uptake is demonstrated in Fig. 2.

#### Automated detection in PSMA PET

**Fig. 3:**
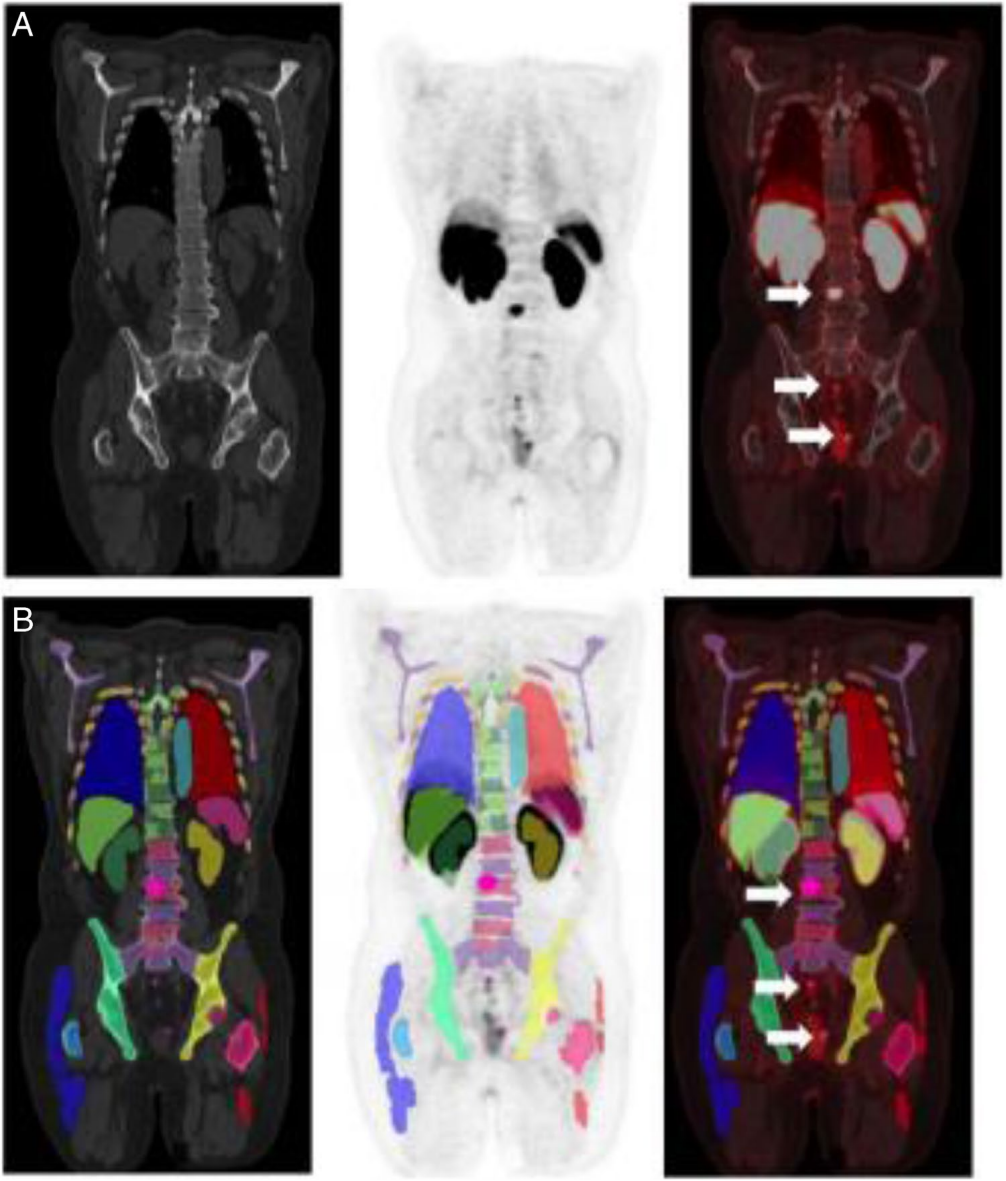
[^18^F]DCFPyL CT, PET, and PET/CT without (**A**) and with (**B**) the deep learning segmentation in low-dose CT of PET/CT. The individual colors represent the respective segmented organs, including the reference organs (liver and aorta). The aPROMISE deep learning algorithm performs automated segmentation of organs, which enables the automated localization, detection and pre-segmentation, and quantification of the potential lesions in PSMA PET/CT. The detection of lesions in prostate is identified by a red hotspot (demarcated in the image by white arrows)

A data set with 235 PSMA PET/CT scans annotated by experienced nuclear medicine readers was used to develop and tune an algorithm for potential lesion detection and segmentation (Supplemental data Table 1). The detection is based on the anatomic segmentations fused to the PET image, and search for potential lesions in bones, lymph nodes, and prostate is done by independently tuned blob detectors [13]. To reduce the number of false positives, a model of normal uptake in the liver, kidneys, and bladder, based on the organ segmentation and the PET intensities, is fitted to the PET image so that this uptake can be suppressed before search for potential lymph node and prostate lesions. To the same end, filtering potential lesions based on SUVmax, SUVmean, uptake volume, and location follows the search. Lesions are segmented by the fast marching method [14], whereby high uptake in proximity to the original blob is included. An illustrative example of lesion detection and segmentation is demonstrated in Fig. 3. From the lesion segmentations, important lesion characteristics can be quantified such as SUVmax, SUVpeak, SUVmean, and uptake volume.

### Statistical methods

The automated organ segmentations were compared to the manual segmentations, and accuracy was evaluated by using Dice score; the mean and its 95% confidence interval were reported. The Dice score between two segmentations, A and B, is a measure of relative overlap and is defined as follows (24):

$$DICE\left(A,B\right)=\frac{2|A\cap B|}{\left|A\right|+|B|}$$

Accuracy and consistency of automated measurements of liver and aorta reference values were evaluated for each reader using intra-reader Pearson correlation and inter-reader standard deviation. For automated lesion pre-segmentation, the sensitivity was evaluated as the percent of manually selected lesions that were automatically pre-segmented by aPROMISE. Intraclass correlation (ICC2) was used to evaluate the quantitative reproducibility of the lesions detected by aPROMISE. As an analytical evaluation study, no prior assumptions were made for the aPROMISE performance to render power calculations. All statistical analyses were made using Python 3.6 with the SciPy library or SPSS Build 1.0.0.1327.

## Results

### Automated CT segmentation

The Dice scores for the organ segmentations are presented in Table 2, and a representative image of segmentations is illustrated in Fig. 1. The average segmentation Dice scores of bone groups ranged from 0.88 to 0.95. The average Dice scores of the reference organs, aorta (blood pool) and liver, were 0.89 and 0.97, respectively. The average Dice scores of prostates and bladder were both observed to be 0.79.

**Table 2:** Segmentation: Dice score of the 14 regions

Segmented organ	Dice score mean (95% CI)	# Evaluated segmentations
Bone—femur	0.95 (0.942, 0.952)	20
Bone—pelvic region	0.95 (0.950, 0.956)	20
Bone—lumbar vertebrae	0.92 (0.910, 0.924)	20
Bone—thoracic vertebrae	0.92 (0.916, 0.925)	20
Bone—thorax	0.88 (0.877, 0.891)	20
Aorta, abdominal part	0.76 (0.727, 0.798)	20
Aorta, thoracic part	0.89 (0.862, 0.917)	20
Kidney, left	0.92 (0.908, 0.948)	20
Kidney, right	0.91 (0.834, 0.982)	20
Liver	0.97 (0.962, 0.968)	20
Lung, left	0.97 (0.956, 0.984)	20
Lung, right	0.98 (0.976, 0.980)	20
Prostate	0.79 (0.716, 0.856)	13
Urinary bladder	0.79 (0.732, 0.865)	19

### Automated reference organ uptake in PSMA PET

The Pearson cross-correlation for blood pool reference values is presented in Table 3 and for the liver reference value in Table 4. It was observed that for blood pool reference values, the correlation is higher between any manual reader and aPROMISE, than between any pair of manual readers. For the liver reference value, the Pearson cross-correlations are higher between manual readers and aPROMISE, than between pairs of manual readers, except between manual reader 1 and manual reader 2. The standard deviations of the reference values across all 89 patients are presented in Table 5 for blood pool and liver. The standard deviation is lowest for the aPROMISE generated reference values for both locations. The increased Pearson correlations together with the decreased standard deviation indicate greater consistency of the automatic reference value estimates, compared to the manually generated values.

**Table 3:** Pearson correlations of aPROMISE against manual reads in quantitative uptake in blood pool (*N* = 89)

	aPROMISE	Manual reader 1	Manual reader 2	Manual reader 3
Manual reader 1	**0.87** (0.80, 0.91)	-	**0.73** (0.62, 0.82)	**0.76** (0.65, 0.84)
Manual reader 2	**0.85** (0.77, 0.90)	**0.73** (0.62, 0.84)	-	**0.78** (0.68, 0.85)
Manual reader 3	**0.82** (0.73, 0.88)	**0.76** (0.80, 0.91)	**0.78** (0.68, 0.85)	-

**Table 4:** Pearson correlations of aPROMISE against manual reads in quantitative uptake in the liver (*N* = 89)

	aPROMISE	Manual reader 1	Manual reader 2	Manual reader 3
Manual reader 1	**0.95** (0.93, 0.97)	-	**0.97** (0.96, 0.98)	**0.79** (0.70, 0.86)
Manual reader 2	**0.95** (0.92, 0.97)	**0.97** (0.96, 0.98)	-	**0.78** (0.68, 0.85)
Manual reader 3	**0.80** (0.72, 0.87)	**0.79** (0.69, 0.86)	**0.78** (0.68, 0.85)	-

**Table 5:** Standard deviation of aPROMISE and manual assessments in reference organs (*N* = 89)

	Blood pool reference (standard deviation)	Liver reference (standard deviation)
aPROMISE	0.21	1.16
Manual reader 1	0.23	1.29
Manual reader 2	0.26	1.21
Manual reader 3	0.24	1.38

### Automated detection in PSMA PET

The performance of the detection and pre-segmentation of lesions, demonstrated as the percent of manually selected lesions also detected by aPROMISE, for each independent reader is displayed in Table 6. The detection sensitivity of the automated algorithm was 91.5% for regional lymph nodes in patients with high risk localized disease, and 90.6% for any lymph nodes and 86.7% for bone in metastatic patients.

**Table 6:** aPROMISE detection and segmentation of region of interest that are determined to be suspicious for metastatic disease

Detection of potential lesions in following disease settings:	Sensitivity (95% CI) of aPROMISE, evaluated considering reader 1–3, as well as all readers, as ground truth, respectively			
	Reader 1	Reader 2	Reader 3	All readers
Cohort A (Regional PSMA positive lymph node lesions)	91.9% (84.1%, 95.5%) *N* = 74	92.7% (81.7%, 97.9%) *N* = 41	90.4% (82.1%, 95.6%) *N* = 73	91.5% (86.9%, 94.9%) *N* = 188
Cohort B low burden (All PSMA-positive lymph node lesions)	94.5% (87.5%, 98.1%) *N* = 73	93.0% (84.2%, 97.6%) *N* = 57	84.7% (75.1%, 91.6%) *N* = 72	90.6% (86.9%, 94.0%) *N* = 202
Cohort B low burden (PSMA-positive bone lesions)	81.0% (69.6%, 89.5%) *N* = 58	91.5% (81,0%, 97.1%) *N* = 47	88.5% (78.8%, 94.7%) *N* = 61	86.7% (81.1%, 91.3%) *N* = 166

The number of false positive lesions detected by aPROMISE evaluated for each reader is presented in supplemental table 2. aPROMISE detected and pre-segmented an average of 19.5 possible regional lymph nodes per patient with high-risk localized disease that the reader did not select. The corresponding numbers for lymph node and bone lesions in metastatic patients were 90.8 and 8.3, respectively. The quantitative reproducibility of SUVmax, SUVpeak, and SUVmean in the pre-segmented lesions were 100% (ICC2 = 1). The overall quantitative performance of aPROMISE assisted-read, including the manually selected lesions, measured as ICC2 was 0.99 for SUVmax and 0.92 for SUVmean.

## Discussion

The increasing availability and use of novel imaging agents within nuclear medicine warrants the development and validation of technology that reliably localizes, segments, and quantifies the specific tracer activity in PET/CT. Additionally, the functional imaging tracers are specific to the biological activity of their respective targets. The biodistribution and pathophysiological uptake of PSMA-targeted imaging tracers is distinct from that of FDG. Our effort has been to apply automated image analysis to tailor anatomical contextualization and potential lesion detection to PSMA PET/CT, with the aim to provide relevant structural information as well as high sensitivity of detecting lesions.

The deployment of automated image analysis systems into routine diagnostic imaging has many potential advantages. First, automation can standardize interpretations thus improving inter-reader agreement in localization and quantitative assessment. Second, automation can improve reader efficiency by reducing time spent evaluating obvious image findings, while simultaneously guiding the human reader’s attention to more challenging, equivocal findings. Third, automation can potentially accelerate the “learning curve” human readers must face when interpretations of new imaging modalities are integrated into routine care. Finally, automated image analysis might be used not only to identify abnormal lesions similar to human readers, but also extract additional diagnostic, prognostic, or predictive information contained in the raw imaging data not otherwise accessible to human readers.

Accurate and consistent anatomical segmentation in CT is essential in medical image analysis and radiation dose planning. The manual segmentation task is mundane, labor intensive, and inherently variable. There have been prior reports on the use of deep learning technology in semantic segmentation of contrast-enhanced or diagnostic CT for image analysis, particularly for application in treatment planning [17–20]. In recent work, Liu C et al. demonstrated a Dice score of 0.85–0.88 for automated prostate segmentation [19]; their work using the contrast enhanced CT achieved performance similar to that observed with MRI imaging in the PROMISE12 challenge [21]. However, the low soft tissue contrast and resolution in low-dose non-contrast-enhanced CT images of PET/CT provide a more difficult challenge in obtaining a clear automated volumetric segmentation of small organs. The performance of our aPROMISE algorithm in prostate segmentation in low-dose CT, without contrast, was similar to that of Nemoto T et al. who also demonstrated a mean Dice score of 0.79 for prostate [22]. The Dice score of the bones and the visceral organ were observed to be 0.88 or above, indicating a much better performance of the algorithm in larger organs. The prostate data does warrant manual review of the prostate segmentation in the aPROMISE analysis of patients with localized disease in PSMA PET/CT.

The first step of aPROMISE, to accurately segment the organs in the low dose CT, enables the subsequent step of quantification in the reference organs of PSMA-ligand PET. PSMA expression in prostate cancer in relation to the reference organs as detected by PSMA ligand PET would standardize quantitative reporting [6]. Notably, quantification of PSMA uptake in PET/CT in relation to liver and blood pool are likely to be critical parameters for selection of patients for PSMA-targeted therapeutics. In ongoing clinical trials, PSMA-positive lesions where SUVmax is above 1 or 1.5 times liver SUVmean have been used as a threshold for selecting patients to be treated with 177Lu-PSMA 617 (NCT03805594) and for 177Lu-PSMA I&T (NCT04297410). Translating such quantitative criteria from clinical trials into clinical practice would require a platform that can provide the consistency of centralized reading at the local level. Our study demonstrates that aPROMISE enables greater reproducibility and higher consistency in reporting the quantitative assessment of reference organs than that of three experienced nuclear medicine physicians.

The overall performance of our methodology in detecting sites of prostate cancer was similar to the recent work by Zhao et al., which employed deep learning for detecting PSMA lesions in the local pelvic area [12]. The independent evaluation of aPROMISE demonstrated that the analytical detection algorithm is proficient in detecting lesions (above 90%) that are manually determined to be pathological in nature. In a recent study [9], a threshold above SUV 4.3 was used for detecting lesions. Had a threshold of SUV=4.3 been used in our study, the detection sensitivity of regional lymph nodes in high-risk localized disease would have dropped from 91.5 to 75.0%, the sensitivity of lymph node metastasis in metastatic disease would have dropped from 90.6 to 76.2%, and the sensitivity of bone metastases in metastatic disease would have dropped from 86.7 to 61.8%. With the lower threshold of SUV=3.0 employed for bone metastases in another study [10], the sensitivity would still have dropped from 86.7 to 77.1%.

The detection and pre-segmentation algorithm demonstrated high sensitivity, also when considering lesions with low uptake. This is beneficial for the reader, decreasing the time spent on segmenting lesions and simultaneously mitigating inter- and intra-reader variability in quantitative assessments. The detection algorithm did however also generate a high number of false positives. The majority of these false positives can be readily disregarded by a reader as they arise in physiological uptake, most notably in the intestines. One can employ CNN for detection and segmentation. However, to successfully train a CNN to account for both soft tissue and bone lesions in uncommon locations, or with unusual uptake patterns, an enormous data set is required. Furthermore, training of CNN will also be tracer specific, so for tracer agnostic detection and pre-segmentation, a large data set comprised of all PSMA tracer will be required. In comparison, our approach of blob detection and fast marching methodology in lesion detection and pre-segmentation has demonstrated a robust solution of whole-body image analysis.

The study also demonstrated disparity of outcome based on reader experience in PSMA imaging. In comparison to his counterparts, reader 2 was consistently conservative in calling the PSMA positive lesions in all tissue types (Table 6). Concurrently, this reader also had very limited experience with PSMA PET/CT. A more trust in automation and in algorithms that have been validated can enhance the consistency of patient diagnosis. We are keen to explore and enhance the relationship of aPROMISE with the physician in real-world practice.

The retrospective design without pre-defined success criteria was a limitation of the current study; however, the objective of the study was to evaluate the performance of the novel platform for its subsequent validation in specific clinical context. The use of three independent and experienced nuclear medicine readers in the evaluation of the aPROMISE algorithms has mitigated some of the risk of bias. The individual organ segmentation is a laborious process, as an example—it takes an estimated 15 to 20 min to volumetrically segment a typical organ in low-dose CT, we were limited in our reliance on the segmentations performed by one experienced reader, and there was no consensus segmentation from multiple readers. Some studies have used overlap of multi-reader segmentations [23]. Such a solution of taking the intersection of multiple readers would result in a truncated volume and not necessarily yield a more accurate standard for comparison against the deep learning algorithm. A limitation in the study design was to not evaluate detection and pre-segmentation of primary tumors in the prostate gland. One of the primary limitations of aPROMISE in analyzing PSMA PET/CT images was the absence of ureter segmentation. The hotspots in the ureter from the physiological uptake in urine are a confounding factor in the assessment of PSMA uptake in lymph nodes in the pelvic area. We are generating labeled data which can enable the algorithm to avoid urine uptake in subsequent versions of the aPROMISE platform.

## Conclusion

The study demonstrated that aPROMISE accurately segments organs in low-dose CT. This segmentation algorithm enables the automated quantification of tracer uptake in reference organs that are more reproducible, and consistent than those obtained manually. Finally, aPROMISE demonstrated high sensitivity in detection and pre-segmentation of regions of interest that are determined to be suspicious for metastatic disease. The efficient and accurate segmentation, localization, detection, and quantification of PSMA PET/CT can facilitate standardized assessment in clinical practice. aPROMISE platform warrants further validation in specific clinical contexts.

## Supplementary Information

Below is the link to the electronic supplementary material.

Supplementary file1 (DOCX 4478 KB)

## Data Availability

Data is proprietary material of Progenics Pharmaceuticals, Inc., USA. The data is available upon request for research use.

## Code availability

Data is proprietary material of EXINI Diagnostics AB. The code is part of CE marked software as a medical device—aPROMISE. The device is available for research or commercial use upon request.
