# Supplementary material for: Analytical performance of aPROMISE: automated anatomic contextualization, detection, and quantification of [18F]DCFPyL (PSMA) imaging for standardized reporting
Source: Eur J Nucl Med Mol Imaging. 2021 Aug 31;49(3):1041–51. doi: 10.1007/s00259-021-05497-8 (PMC8803714; doi:10.1007/s00259-021-05497-8)
Supplement: Supplementary file 1 — Supplementary file1 (DOCX 4478 KB) [file 259_2021_5497_MOESM1_ESM.docx]

**Analytical Performance of aPROMISE: Automated Anatomic Contextualization, Detection and Quantification of [^18^F]DCFPyL (PSMA) Imaging for Standardized Reporting**

**Authors**: Kerstin Johnsson^1^, Johan Brynolfsson^1^, Hannicka Sahlstedt ^1^, Nicholas G. Nickols^2,3,4,5^, Matthew Rettig^4,5,6^, Stephan Probst^7^, Michael J. Morris^8,9^, Anders Bjartell^10^, Mathias Eiber^11^, Aseem Anand^1,8,10^

^1^Department of Data Science and Machine Learning, EXINI Diagnostics AB, Lund, Sweden; ^2^Radiation Oncology Service, VA Greater Los Angeles Healthcare System, Los Angeles, CA; ^3^Department of Radiation Oncology, David Geffen School of Medicine, University of California Los Angeles; Los Angeles, CA; ^4^Department of Urology, David Geffen School of Medicine, University of California Los Angeles; Los Angeles, CA; ^5^Institute of Urologic Oncology, Jonsson Comprehensive Cancer Center, University of California Los Angeles; Los Angeles, CA_;_ ^6^Division of Hematology-Oncology, VA Greater Los Angeles Healthcare System, Los Angeles, CA. ^7^Nuclear Medicine, Medical Imaging, Jewish General Hospital, McGill University, Montreal, QC, Canada_;_ ^8^Department of Medicine, Memorial Sloan Kettering Cancer Center, New York, USA. ^9^Weill Cornell Medical College, New York, USA. ^10^Department of Translational Medicine, Division of Urological Cancers, Malmö, Lund University, Lund, Sweden.^11^Department of Nuclear Medicine, Klinikum Rechts der Isar, Technical University of Munich, Munich, Germany.

**Corresponding Author**

Aseem Anand, PhD

Department of Translational Medicine, Division of Urological Cancer, Lund University

Waldenströms gata 5, SE 205 02 Malmö, Sweden

E-mail: [aseem.anand@med.lu.se](mailto:aseem.anand@med.lu.se)

Phone: +46706604084; Fax: +4640336911

**Supplemental Data**

**1A**


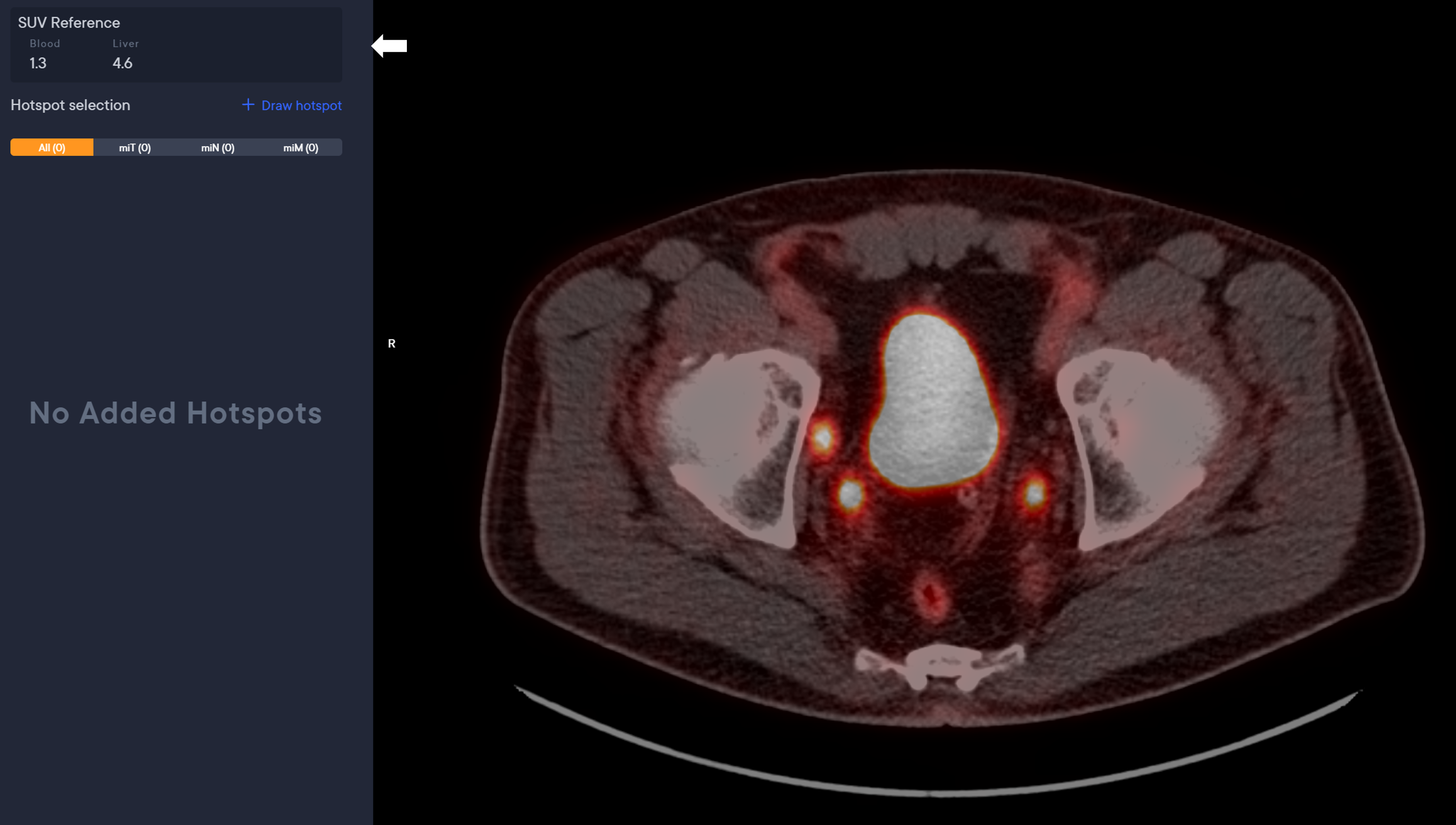


**1B**


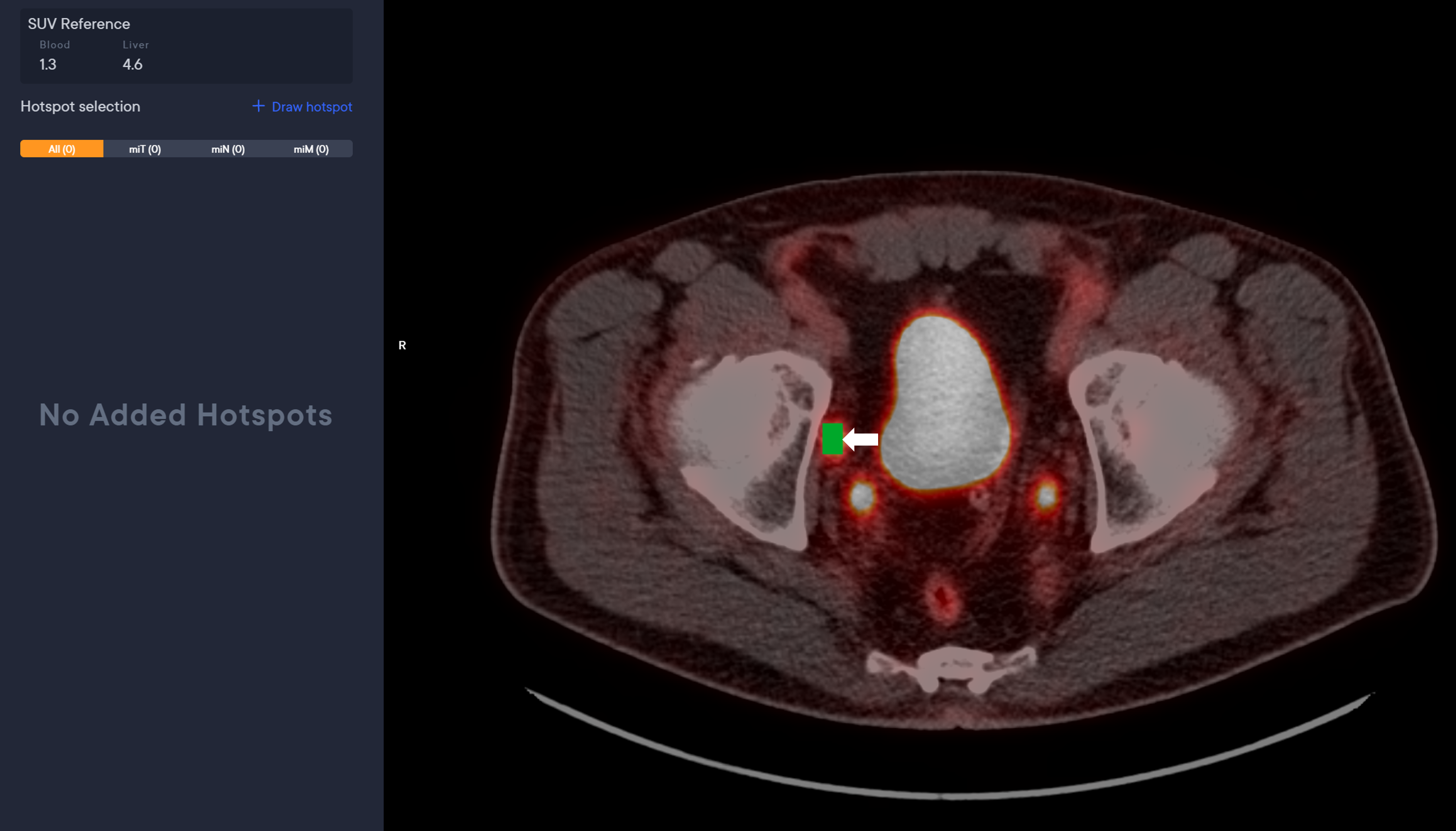


**1C**


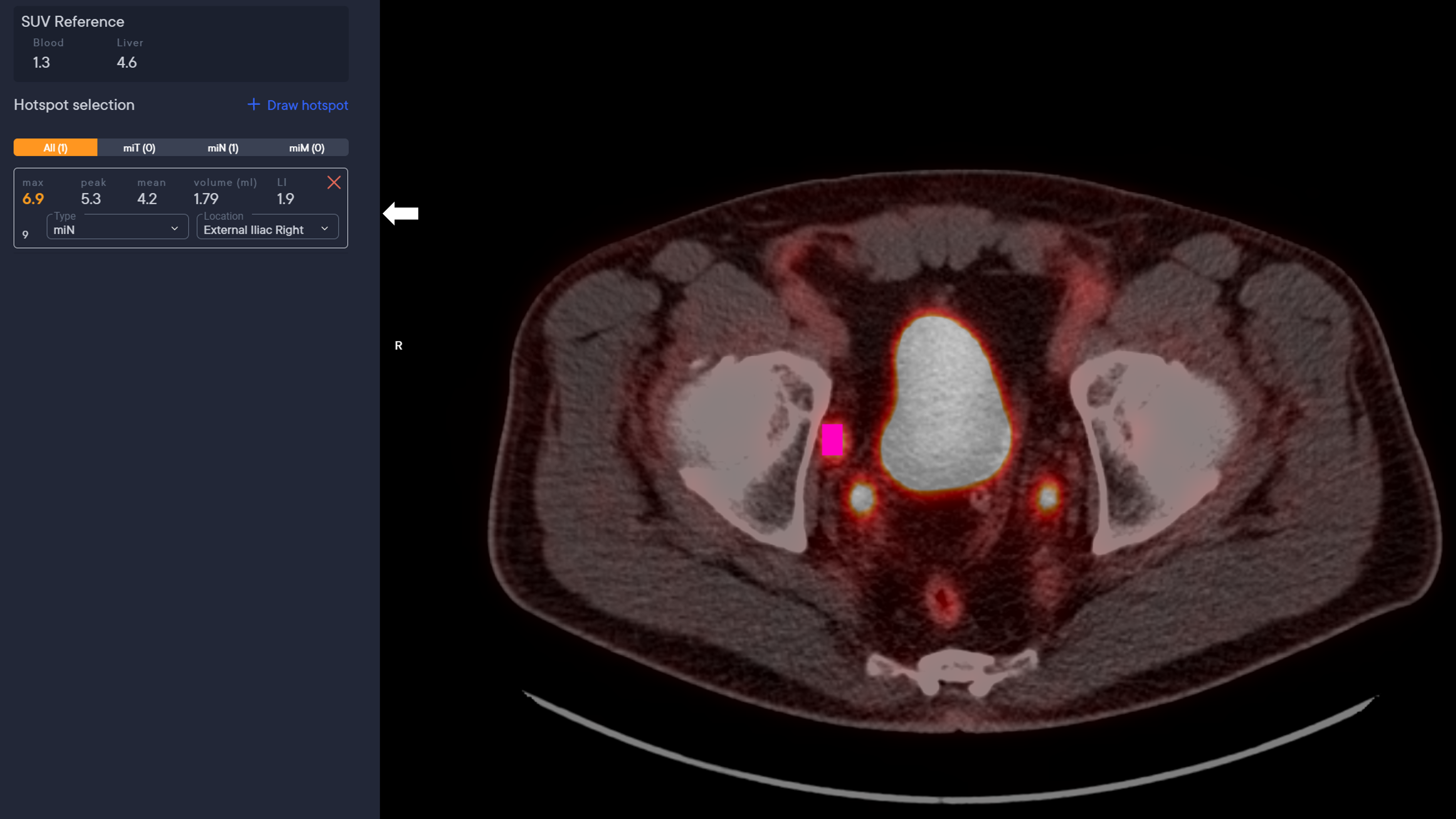


**SUPPLEMENTAL FIGURE 1.** The illustration of aPROMISE workflow, the physician is provided with the automated reference values for blood pool and liver (white arrow), no hotspots list is pre-determined for the physician (1A). Upon physician’s review and hovering over the hotspots the pre-detected and segmentation is highlighted as green (1B). Physician can either select the pre-segmented hotspot or draw his own. The localization, staging and quantification (white arrow) are automated when physician makes the selection (1C).

**SUPPLEMENTAL TABLE 1.** Data used for algorithm development is listed in the table below.

| **Objective** | **Training and Tuning Data** | **Design** |
| --- | --- | --- |
| **To develop Convolutional Neural Network (CNN) algorithm for segmentation of organs in low dose CT.** | Low dose CT images from ^18^F-FDG PET/CT examinations from Sahlgrenska University Hospital - (2016/103)  N=184 | Manual segmentations by a team of experienced nuclear medicine physician of bone and soft tissue organs in low dose CT were used to train CNNs for automated organ segmentation. |
|  | Low dose CT images from 99mTc-MIP-1404 SPECT/CT scans from Ph II clinical trial (NCT0261506) of localized prostate cancer patients  N=62 |  |
| **To develop blob detection and fast marching segmentation algorithms for detection and pre-segmenting hotspots in PSMA PET** | [^18^F]DCFPyL PET/CT scans from Investigational studies of metastatic prostate cancer patients under PyL Research Access Program from Jewish General Hospital and John Hopkins (IND #121064).  N=235 | Manually detected and segmented PSMA lesions by two experienced nuclear medicine physicians (both with >5 years of experience with PSMA tracers) were used to optimize blob detection and fast marching segmentation algorithms to detect and pre-segment potential PSMA lesions. |

**SUPPLEMENTAL TABLE 2.** Number of false positive lesions per patient by aPROMISE.

| **Detection of potential lesions in following disease settings:** | **Average number of false positive lesions per patient (95% CI), i.e., possible lesions detected by aPROMISE not selected by the reader.** | | | |
| --- | --- | --- | --- | --- |
|  | **Reader 1** | **Reader 2** | **Reader 3** | **All Readers** |
| **Cohort A**  (Regional PSMA-positive lymph node lesions) | 19.4  (17.7, 21.1) | 19.7  (18.0, 21.3) | 19.4  (17.7, 21.1) | 19.5  (18.6, 20.5) |
| **Cohort B**  (All PSMA-positive lymph node lesions) | 90.2  (80.8, 99.6) | 90.8  (81.4, 100.2) | 90.5  (81.1, 99.9) | 90.5  (85.1, 95.9) |
| **Cohort B**  (PSMA positive bone lesions) | 8.2  (3.2, 13.1) | 8.3  (3.3, 13.2) | 8.0  (3.1, 13.0) | 8.2  (5.3, 11.0) |
